# Being enjoyably challenged is the key to an enjoyable gaming experience: an experimental approach in a first-person shooter game

**DOI:** 10.1080/20009011.2018.1474668

**Published:** 2018-05-15

**Authors:** Anne Corcos

**Affiliations:** ^a^ CURAPP-ESS UMR 7319, CNRS, Université de Picardie Jules Verne, Amiens, France

**Keywords:** Flow theory, first-person shooter game, gamer experience, challenge, concentration, enjoyment, self-rewarding experience

## Abstract

Applied to video games, Csikszentmihalyi’s work on flow evidences that a positive gaming experience is intrinsically self-rewarding and primarily determined by the skill/challenge balance. A multi-layered measure of enjoyment is built to take these components into account. Gamers were asked to report the concentration-enjoyment they experienced during a first-person shooter game, and to better assess the gap between skill and challenge, the challenge enjoyment was also rated. Along with concentration level, concentration enjoyment is used to build a gaming experience typology that accounts for the self-rewarding component. An enjoyment-based challenge mapping is also drawn up, crossing challenge enjoyment and challenge level. The results show that this integrative enjoyment measure strengthens the causal link between challenge and gaming experience. Most importantly, the findings suggest that challenge or concentration-based enjoyment measures outweigh the standard concentration and difficulty measures as they are more likely to ensure a pleasant and positive experience (flow or relaxation) for the gamers. Indeed, *regardless of the reported level of challenge*, a gamer is more likely to have a positive experience when challenged at a level she perceives as pleasant. This article emphasizes the importance for game publishers of gathering enjoyment-based concentration and challenge assessments to ensure a positive gaming experience and gamers’ commitment.

## Introduction

Based on Csikszentmihalyi’s [1] work, a great deal of research has been carried out to corroborate the flow approach and, more generally, to study the interplay between game difficulty and player experience (XP). This paper follows this line of research, focusing however on the enjoyment part of flow theory. Its originality lies in the fact that it emphasizes two elements which have been given limited attention in the literature so far: first, for a gaming experience to be positive *it has to be self-rewarding* regardless of the outcome of the game, and second, a necessary condition for the skills and challenge to be balanced is that *the gamer needs to enjoy the challenge level*. To account for these features, we use a multi-dimensional measure of the enjoyment experienced during the game. The self-rewarding dimension of the experience is assessed using a self-reported measure of concentration enjoyment. It aims at appraising to what extent individuals enjoy the state of concentration the game puts them in. In the same way, a self-reported measure of difficulty enjoyment is collected to ensure that the challenge level remains pleasant for the gamer.

To this aim, I use Orero et al.’s data [2] in which subjects had to play a highly immersive first-person shooter game (FPS).^1^ First, using the gamer’s self-reported concentration enjoyment along with her concentration level, we build a gaming experience classification accounting for its self-rewarding component. In a standard way, the classification ranges from boredom to flow, to anxiety and relaxation. Second, the self-reported difficulty enjoyment measure is crossed with the subjective difficulty assessment to estimate the frontier of an enjoyable challenge. Altogether, the two-step enjoyment measure makes the challenge-XP analytical framework more efficient and relevant to explain and predict the gaming experience.

The originality of this study is that it broadens the definition of gamer enjoyment by encompassing reflexive measures of the pleasure induced by the concentration and the challenge faced while playing. This analytical framework is of interest for several reasons.

First, it makes it possible to deepen our understanding of the player’s experience. The need for the gaming experience to be self-rewarding is not captured by standard approaches which actually do not aim at measuring this dimension. Moreover, the self-rewarding property of the gamer experience is not easy to grasp as the standard measure of game enjoyment can be biased by a confounding factor: game outcome. Besides, a highly concentrated gaming episode does not necessarily result in a positive experience.

Moreover, the pleasure of being challenged might enable to more accurately estimate the subjective frontier between balanced and unbalanced (under and over) challenging situations than any comparison between objective and subjective measures of the game difficulty level. Besides, an analysis which disentangles the challenge from the challenge enjoyment enables for the interplay between challenge and enjoyment not to be a one-to-one relationship as in [3]. Indeed, although being challenged is a desirable state, an over-challenging game could undermine the challenge enjoyment.

Ultimately, the two-step enjoyment measure might improve the Challenge-XP causal model. One may even wonder whether reflexive enjoyment measures may determine the player’s experience even more than either the challenge or concentration levels. Indeed, the skill-challenge balance and concentration are basically only a means of providing enjoyment to players whose primary goal is precisely to have fun. There are reasons to believe that a leading indicator of game experience should be based on the enjoyment component of challenge and concentration rather than on their absolute levels. If this feature was borne out by the data, game publishers would be well-advised to set up an enjoyment measure to assess these two neglected sides of gaming enjoyment.

Beyond enriching the understanding of the link between challenge and XPs, this research could improve our understanding of what defines and promotes a state of flow and could benefit to fields such as learning, education, or commitment in a job.

## State of the art

Video games are proving to be very popular with 2.2 billion casual or regular gamers across the globe in 2017.^2^ Many studies [4–7] have examined so far the reasons why people play video games, which include the desire for competition, challenge, social interactions, entertainment, excitement, and escape [8]. However, the very primary goal of games is enjoyment. Fang et al. [9] seek to define and measure enjoyment during a game, while Lin et al. [10] or Klimmt et al. [11], Klimmt et al. [12], and Klimmt et al. [13] study the conditions of its occurrence (see [14] for a review of quantitative studies on enjoyment in video games). Vorderer et al. [15] endeavor to define a theoretical framework of enjoyment for games.

Video games have also been given special attention because of their ability to completely and pleasantly immerse players in their activity. Csikszentmihalyi’s [1] seminal work precisely focuses on the flow experience of individuals intensively immersed in *self-rewarding* activities, whether in leisure or at work. His research is specifically devoted to characterizing this experience and to better understanding its proximal conditions. Csikszentmihalyi shows that under-used skills may lead to boredom just as over-used skills may result in anxiety. By contrast, the flow optimal experience, characterized by a self-rewarding immersion state, occurs when challenge and skills balance out, and an individual’s skills are strongly stretched.

Csikszentmihalyi’s research on flow and Sweetser and Wyeth’s adaptation to games [16] make it possible to characterize the optimal gamer experience, as well as its proximal conditions. The game must provide proximal goals and immediate feedback (close and connected to the goals) perceived as rewards, and it should enable progress. The game, which should be completable, must promote the individual’s sense of control over her actions. All these elements are purposely part of FPS games and, using them as an analytical framework enables to control for the necessary conditions of the optimal gamer experience to be met, and to focus on issues of concentration and challenge.

Based on Csikszentmihalyi’s Flow Theory, a large literature has studied how enjoyment, immersion, and challenge link to provide the gamer with flow experience ([17,18] in FPS). Jennett et al. [19] have studied the characteristics of the game (pace, interaction with other players on a website, realism, and easier identification with the shooter in a first-person game) fostering gamer immersion and enjoyment. Brockmyer et al. [20] characterize the experience by its immersive dimension, and aim at assessing game engagement (see [21] for a review on engagement in digital games). Jegers [22] emphasizes instead the association between flow and enjoyment, while Cox et al. [23] examine the role of challenge in immersion. Other studies (see [3]) focus on the link between challenge and enjoyment.

Applied to video games, Csikszentmihalyi’s work makes it clear that the gaming experience deeply relies on an accurate estimation of the difficulty-skill balance. Nevertheless, while an appropriate challenge level appears to be the cornerstone of Csikszentmihalyi’s flow experience, its standard estimation often reveals significant drawbacks. The perceived difficulty level is obtained using questionnaires (to what extent did you find the game difficult?) whose answers are collected after the game session [3]. The objective difficulty level of a game is determined using objective difficulty level assessment (such as game publishers’ evaluation), as well as gamers’ rankings or relative measures (comparing a gamer’s ranking with her competitors’). The challenge level (as in [3]) is deemed to stem from the comparison between these measures. However, this whole process only provides an imperfect estimate of the boundary where skills and difficulty balance out. It does not enable to ascertain whether the gamer’s skills were *truly* over- or under-used during the game. When a gamer perceives a game as difficult, whether she is a beginner or an expert, it does not necessarily imply that it *over*-challenges her skills. Since the gaming experience critically depends on the difficulty-level departure from the gamer’s skills, any uncertainty in the discrepancy is likely to undermine the ability to infer the gamer experience. The frontier between adequate and over-stretched skills lies actually where the gamer *stops enjoying the challenge*. Assessing the locus of points where challenge and gamer skill balance out addresses therefore a major issue whose answer lies partially in the pleasure arising from the challenge.

Csikszentmihalyi’s work also strongly stresses that for the gamer to experience flow, not only does the game have to stretch the gamer’s skills, but the gaming experience must also be self-rewarding. However, the common feature of the studies mentioned so far is that they share a narrow view of game enjoyment, which is mostly based on the pleasure gained from the game as measured through self-reported assessment,^3^ GEQ questionnaires [24], or physiological measures [2,18,25,26]. Two drawbacks can also be mentioned here when using questionnaires. There may be confounding factors of enjoyment, such as outcome, which distort perceived enjoyment [3,27]. There is indeed some concern that a winner enjoys the game more than a loser. Besides, these measures do not embed a central feature of the flow experience that must be self-rewarding. *Being immersed must be intrinsically pleasant* could characterize autotelic tasks and define Csikszentmihalyi’s flow state. Hence, although a high concentration level is required to experience flow, it is not a sufficient condition. A broadened understanding of enjoyment encompassing concentration enjoyment would hence account for the self-rewarding dimension of the concentration state. Such a measure would also allow to overcome the confounding issue between game performance and game enjoyment.

Considering these challenge- and concentration-based enjoyment components, we aim at both characterizing gamer experience during an FPS game and understanding its antecedents. My findings underline the suitability of the use of enjoyment-based measures to assess the challenge-XP relationship. They could even outperform standard measures of concentration and challenge: Indeed, the pleasant dimension of the challenge determines the pleasant nature of the player’s experience in two-thirds of the cases. Regardless of either the challenge or concentration level, being enjoyably challenged appears therefore to be the very key to an enjoyable gaming experience.

The next section outlines the experimental protocol; Section 3 presents the analytical framework, while Section 4 sets out the results. Section 5 concludes.

### The experimental setup^4^

In Orero et al.’s experiment [2], 42 players aged between 18 and 39 played 4 rounds of the Halo3 game. All the sessions would start with an introductory round (Introduction Round) corresponding to the first minutes of the game. After this first round, participants would play three more rounds, whose presentation order was counterbalanced to avoid any order effect. According to experts, the games Tank, Sniper, and Difficult were increasingly challenging (objective difficulty assessment).

After each round, the participants filled out a set of evaluations on a six-point ranking scale. They were asked to rate the level of difficulty experienced during the round (subjective Difficulty Assessment, DA), as well as the pleasure gained from the difficulty encountered (Difficulty Enjoyment, DE). The same was asked regarding their concentration level (Concentration Assessment, CA), and concentration enjoyment (CE).^5^

Finally, to gather different layers of players’ experiences, at the end of the experiment (after the participants had completed the four rounds), participants had to answer two questions (comparative self-reported measures) where a comparison between the four rounds was proposed as follows: Which round was the most (least) difficult?

## The analytical framework

The experiment has been designed to collect layers of enjoyment that link which each step of the gamer’s experience. First, along with concentration assessment (CA), the concentration enjoyment (CE) measure seeks to characterize the player’s experience *accounting for its self-rewarding dimension*. For its part, combined with the subjective measure of difficulty DA, the measure of difficulty enjoyment DE is expected to ensure that the player’s skills are challenged in a way that *remains pleasant*.

### The concentration enjoyment measure as a proxy of the self-rewarding component of gamer experience

According to Csikszentmihalyi [1], the flow state is intrinsically rewarding, making experience enjoyment a key factor to define the gaming experience. Not only does the rewarding experience property require the game to be enjoyable, but the ‘intrinsically’ rewarding dimension primarily emphasizes the need for the pleasure to derive from the gamer’s mental state rather than from the outcome of the game.

Yet studies do not seem to give this point the weight it deserves, as the standard subjective self-reported measure of concentration CA does not intend to capture the enjoyment gained from the gamer’s mental state: high concentration does not necessarily involve high concentration enjoyment nor does a low concentration level result in an unpleasant gaming experience.

To fit with the rewarding requirement for an experience to be optimal, the gamer has to be concentrated and to *enjoy being concentrated*. Therefore, to better characterize the gaming experience, the concentration measure should be complemented by an assessment of the pleasure of being immersed (CE), which this experiment purposely captures. Together, concentration assessment and concentration enjoyment measures make it possible to disentangle immersive and non-immersive experiences, but also pleasant and unpleasant ones.

Four gaming experiences (boredom, relaxation, flow, and anxiety) out of the eight pointed out by Nakamura and Csikszentmihalyi (2014) are obtained by crossing CA and CE. Table 1 below shows the correspondence between the two-dimensional concentration measure and XPs, where low (CA or CE≤4) and high (CA or CE>4) levels of concentration measures have been distinguished.

**Table 1.** Gaming experience classification accounting for the self-rewarding dimension.

		Concentration-level assessment	
		CA_L_	CA_H_
Concentration enjoyment	CE_L_	Boredom	Anxiety
	CE_H_	Relaxation	Flow

H and L subscripts stand for high and low levels of CA and CE.

In this perspective of a self-rewarding experience, the flow experience can now be understood as a high *and* pleasant concentration state (high CA and CE). At the other end of the spectrum, individuals who report low levels of CA and CE are not concentrated, and they do not enjoy this lack of concentration. They are deemed to experience boredom (B). Intermediate states lie between boredom and flow. Anxious individuals (A) perceive their high level of concentration as unpleasant (CA_H_ and CE_L_), pointing to an excessive concentration level, while relaxed ones (R), although not very concentrated, enjoy this low concentration level (CA_L_ and CE_H_).

### DE is relevant to assess whether skills fit with the challenge level

Consistent with Csikszentmihalyi’s resarch, the gaming experience is primarily determined by the challenge level of the game, which is usually assessed comparing the game difficulty assessment with the gamer’s skills. This standard approach has shown several weaknesses so far. Confronting the gamer’s expertise with an expert’s objective ranking only provides a weak proxy of the challenge the gamer actually faces. First, the balance between the objective difficulty ranking and skills is subjective and task dependent. It can therefore not easily be grasped by comparing these external measures. Second, the gamer’s experience is defined at least as much by the difficulty level of the game as by the enjoyment in being challenged. Therefore, neither the objective measure of difficulty (by an expert) nor the subjective one (difficulty assessment, DA) enables to appraise the pleasure aroused by this difficulty.

The DE measure of how pleasant the difficulty level was can be used to disentangle pleasant and unpleasant challenging games: gamers report a low CE level (DE_L_) when either over- or under-challenged. On the contrary, situations where the gamer is comfortable with the difficulty level of the round present a high DE level (DE_H_).^6^ A high DE therefore characterizes pleasant gaming experiences, whether challenging or not. For its part, the subjective difficulty level assessment measure points out whether skills are stretched (DA_H_) or not (DA_L_).

**Figure 1.**
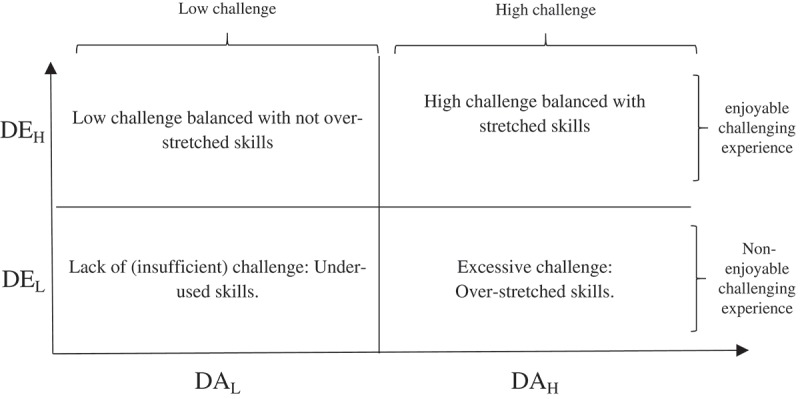
Enjoyment-based challenge mapping.

Crossing these two dimensions provides enjoyment-based challenge mapping (see Figure 1) that ranges from the least challenging situation (low DA & DE) to the most (even disturbing) challenging (high DA and low DE). Intermediate levels are more pleasant (high DE) with the comfortable situation (low DA) being distinguished from the challenging one (high DA).

Then, along with DA, the difficulty enjoyment measure makes it possible to assess the skill/difficulty balance and to disentangle high (even too) challenging situations from low (even insufficiently) challenging ones.

### How do enjoyment measures fit in with the causal relationship between xps and challenge?

**Figure 2.**
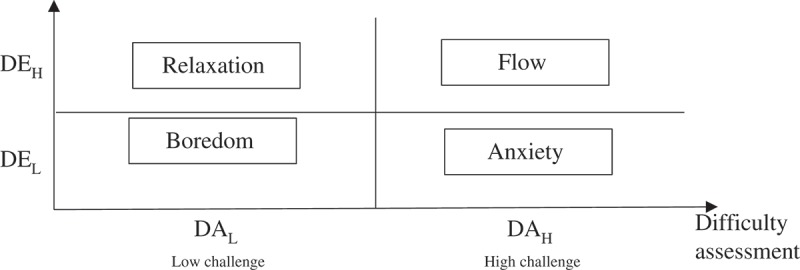
Gaming experience/challenge correspondence mapping.

This section addresses the issue of challenge-XP association and its links with (concentration and difficulty) enjoyment measures. Based on Csikszentmihalyi’s approach, the enjoyment-based challenge-XP association examines how, accounting for enjoyment components, the challenging dimension of the game determines the gaming experience. As Ellis et al. [28], we find a 4-channel model which is close to Csikszentmihalyi’s reduced model (see Figure 2).

Two sets of gaming experiences can be associated with an enjoyable challenge level (DE_H_). First, according to Nakamura and Csikszentmihalyi [29], one major proximal condition to flow experience is being adequately challenged, with gamers’ skills being stretched but not overestimated (DA_H_&DE_H_). However, in this ‘enjoyable zone’ also lies the relaxation state characterized by a pleasant gaming experience, with the skills not being stretched (DA_L_&DE_H_).

Below the enjoyable-challenge area (DE_L_) are unpleasant challenge-level games. An over-challenging game (DA_H_) is prone to bring on anxiety (‘a region of anxiety as challenge increasingly exceeded capacities for action’), as well as an under-challenging one (DA_H_) causes boredom (‘a region of boredom as opportunities for action relative to skills dropped off’(Nakamura and Csikszentmihalyi ([29], p.94)).

### Three-step validation

For the two-step enjoyment measure to be relevant, the three following propositions have to be corroborated by the data:

P1- For the concentration enjoyment measure to be a proxy of the self-rewarding component of the gaming experience, CE and CA must work together: a high level of concentration is not expected to necessarily provide an enjoyable experience, nor is a low level of concentration always expected to involve an unpleasant gaming experience.

P2- Similarly, difficulty enjoyment should not be able to perfectly predict the difficulty assessment (whether objective or subjective), nor should it be inferred from it. Such features support the joint use of DA and DE to point out enjoyably challenging games, with skills stretched appropriately.

P3- In accordance with Figure 2, the predictions related to the challenge-XP causal model embedding the multi-layered enjoyment measure entail that DA and DE properly predict gamer experience:

P3-a:

- High DA and DE are expected to enhance the likelihood for the gamer to experience flow
- Low DA and DE should make a boredom experience more likely
- High DA and low DE are likely to increase the probability of an anxiety experience
- Low DA and high DE should generate a relaxation state.

For the XP-challenge link to be a one-to-one relationship, the reverse proposition is also expected to be true: DA and DE levels should be derived from gamer experience. This can be expressed in the following way, where superscript letters R, F, A, and B refer to XPs (Relaxation, Flow, Anxiety, and Boredom):

P3-b:

- Gamers experiencing Flow (resp. Anxiety) should have higher reported difficulty levels (DA) than gamers in a relaxation state (resp. Boredom): H3-b1: DA^R^<DA^F^ (resp. H3-b2: DA^B^<DA^A^).
- Gamers experiencing Flow (resp. Relaxation) should face higher difficulty enjoyment (DE) than anxious gamers (resp. Bored): H3-b3: DE^F^>DE^A^ (resp. H3-b4: DE^R^>DE^B^).

Provided that both P3 conditions are borne out by the data, the multi-layered enjoyment enables to better assess gamer experience than DA and CA alone.

## Results

The next sections discuss the results and the three-step validation of the analytical framework thoroughly.

### P1- the added value of the concentration enjoyment measure over the concentration assessment measure alone: CE as a proxy of the self-rewarding component of gamer experience

Table 3 below gives the breakdown of the four rounds according to the two-dimensional measure of concentration.

**Table 2.** Round breakdown according to enjoyment-based challenge mapping.

		Challenge mapping						
		Low difficulty assessment (DA_L_)			High difficulty assessment (DA_H_)			
Rounds		Low difficulty enjoyment DE_L_	High difficulty enjoyment DE_H_	Total DA_L_ (a)	Low difficulty enjoyment DE_L_	High difficulty enjoyment DE_H_	Total DA_H_ (b)	N
Introduction	(1)	13	24	**37**	0	5	**5**	42
Tank	(2)	9	23	**32**	4	6	**10**	42
Sniper	(3)	12	18	**30**	4	8	**12**	42
Difficult	(4)	0	0	**0**	22	20	**42**	42
Total	(5)	34	65	**99**	30	39	**69**	168

χ^2^_challenge&round_ = 88.938 (p-value = 0.000) N: number of observations

**Table 3.** Round breakdown according to XPs.

	XPs				
Rounds	Boredom (CA_L_, CE_L_)	Relaxation (CA_L_, CE_H_)	Flow (CA_H_, CE_H_)	Anxiety (CA_H_, CE_L_)	N
(1) Introduction	**24**	14	4	0	42
(2) Tank	15	**14**	10	3	42
(3) Sniper	9	6	**20**	7	42
(4) Difficult	5	1	23	**13**	42
(5) Total	53	35	57	23	168

N: number of observations χ^2^_XPs&round_ = 62.29 (p-value = 0.000)

Line [5] in Table 3 shows that a highly immersive gaming experience does not mean that it is self-rewarding, nor is a low concentration experience necessarily unpleasant. The 88 low attention-demanding experiences (CA_L_) split between boredom (53) and relaxation (35), so do the 80 highly attention-demanding rounds (CA_H_), which can result in either flow (57) or anxiety [23] experiences.

**Observation 1**: Supporting P1, the concentration assessment alone seems to provide only limited information on the deep gaming experience while, combined with CE, it brings out the whole picture allowing to characterize XPs. These results stress the need for a concentration enjoyment measure, and support the relevance of a measure encompassing the self-rewarding dimension of gaming experiences.

### P2- difficulty enjoyment: a measure as critical as the objective and subjective measures of the difficulty level

As Table 3 with concentration measures, Table 2 below provides the breakdown of the four rounds (168 = 42x4 observations) according to the two-dimensional measure of difficulty. We consider both objective (by an expert) and self-reported difficulty assessment measures. We also examine the answers to the comparative questions.

The data have a strong internal and external consistency, the objectively most and least difficult rounds being perceived as such by the gamers both in the comparative questions (‘which round was the most (or least) difficult?’) and the subjective difficulty assessment measures (DA).^7^

The same conclusions can be drawn from the comparison between the objective difficulty measure and DA. As already stated, Introduction, Tank, Sniper, and Difficult rounds have been ranked by experts in ascending order of difficulty. This order has been confirmed by the participants’ self-evaluation, with the average DA increasing with the objective difficulty level. As shown in col. (a) and (b) in Table 2, the two easiest games objectively (Introduction and Tank) are given low DA in 82% of cases. In the same way, the two most difficult rounds objectively (Sniper and Difficult) are perceived as difficult ones (DA_H_) in more than 64% of cases. Overall, the objective difficulty level of the rounds fits with the gamers’ perceptions.

Although these results highlight the very strong consistency between the difficulty level measures, line [5] in Table 2 makes it apparent that, on the contrary, DA and DE converge only in less than 1 out of 2 cases (43%) with a significant but negative Pearson coefficient.^8^ ^,^ ^9^ Only 56% of the rounds rated as difficult (high DA) result in an enjoyable difficulty level (high DE), compared with 65% of the rounds perceived as easy (low DA).^10^ Conversely, DE does not act as a leading indicator of DA. 104 pleasant rounds (DE_H_) come from either high or low DA. 64 rounds characterized by unpleasant experiences (DE_L_) include subjectively easy (DA_L_) and difficult (DA_H_) rounds.

This internal contradiction observed between the difficulty measures DA and DE is in accordance with Abuhamdeh and Csikszentmihalyi’s [3] findings related to the curvilinear relationship between enjoyment and challenge. The value of using such a difficulty enjoyment measure is rooted in these divergences: DE does not boil down to DA but acts rather in a complementary way. DE makes it possible to disentangle feelings that cannot be differentiated when using either expert evaluation, DA, or both.

Furthermore, strikingly, out of the 104 enjoyable challenging experiences (DE_H_), almost two-thirds (62%) are driven by games reported as easy (DA_L_). It raises therefore the question of what comes first in the player’s experience: might the pleasure of being challenged prevail over the challenge level itself? So far, emphasis has been put on the latter (whether objective or subjective), while evidence suggests that DE could perform even better than DA to monitor game enjoyment.

**Observation 2**: Data provide strong support for the relevance of challenge mapping that accounts for difficulty enjoyment. First, the three measures of difficulty (DA, objective, and comparative questions) feature strong consistency. Second, in accordance with P2, the DE measure takes on its full meaning as none of these three measures make it possible to infer the pleasure arising from this difficulty. The role assigned to DE, i.e. untangling over- and under-challenging experiences from those which pleasantly stretch the gamers’ skills, is therefore borne out by the data. Third, knowing DE could be even more relevant than DA, as more than two-thirds of enjoyable challenging rounds are triggered by (self-reported) low challenging games.

### P3-what does multi-layered enjoyment teach us about the causal relationship between xps and challenge?

Several features provide some support to the causal model based on the multi-composite enjoyment measure that links gaming experiences to challenge mapping.

#### The objective difficulty measure can be inferred from the gaming experience

When the gaming experience typology accounts for a self-rewarding component, the objective difficulty level can properly be derived from the gamer’s experience. As shown in Table 3, XPs strongly correlate with the objective challenging level of the rounds: more than three-quarters (76%) of the boredom and relaxation states are experienced while playing the two easiest games (Introduction and Tank), whereas almost 80% of anxiety and flow experiences arise with the most challenging games (Sniper and Difficult).^11^

A more detailed analysis of Table 3 shows that bored gamers are proportionally more numerous in the Introduction game, while anxious ones are mainly found in the Difficult round. Similarly, the relaxation state arises mostly during the two easiest games, while flow is experienced while playing the two difficult ones.

As gaming experiences move from boredom to anxiety, to relaxation and flow, the objective difficulty of the game increases. **Therefore, CE enables the objective difficulty level to be derived from gamer experience.**

However, the reverse does not hold, and low and high challenging rounds are just as likely to cause a self-rewarding gaming experience (CE_H_). Indeed, although the objective level of difficulty shapes the player’s experience, it does not address the pleasure gained by the gamer while concentrated. Table 3 above shows that regardless of CA, concentration enjoyment cannot be predicted based on the level of difficulty: surprisingly, the easiest game (Introduction) triggers almost as many positive experiences (18 relaxation or flow) as negative experiences (24 boredom or anxiety). Similarly, the Difficult round elicited a rewarding experience (relaxation or flow) for 24 gamers and a negative one (boredom or anxiety) for 18 players.

**Observation 3**: The CE influence from the gaming experience on DA is only a one-way relationship: the gaming experience typology encompassing a self-rewarding dimension makes it possible to infer the objective difficulty faced by the gamer. The reverse is not true as a weakly challenging game is as prone to result in a self-rewarding experience as a highly challenging one.

There is therefore a need to better predict the gaming experience and to understand what makes it pleasant. The subjective measure of difficulty enjoyment might conveniently fulfil this role.

#### The two-dimensional difficulty measure (DA and DE) further enhances our understanding of the interplay between challenge and gamer experience

**Figure 3.**
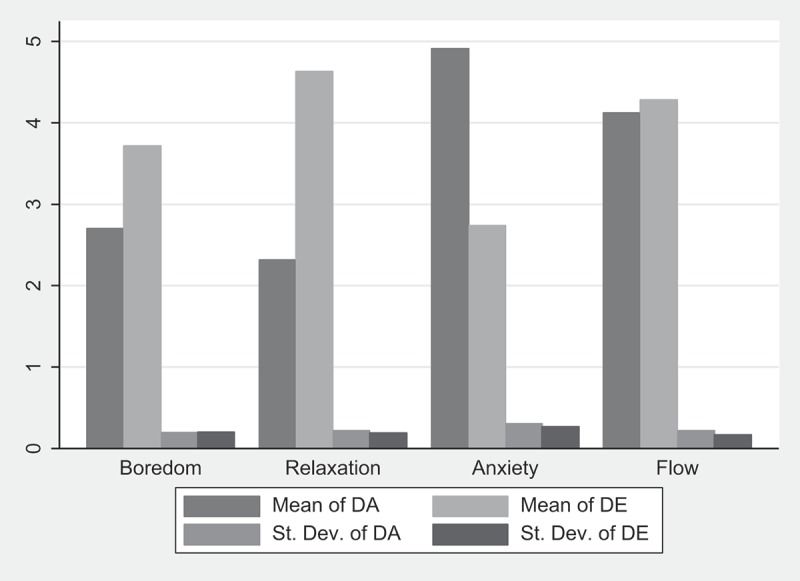
DA and DE averages over XP.

Figure 3 below displays the average values of DA and DE and their standard deviation, according to XP. Tables 4 and 5 below provide the Student mean tests of the difficulty comparison across XPs.^12^ Table 4 compares the difficulty assessment (DA), and Table 5, the difficulty enjoyment measures (DE). In each cell are provided the difference between XPs under comparison, the value of the test, as well as its p-value in parentheses.

**Table 4.** Student mean tests of DA comparisons.

Hypotheses	XPs under comparison	ΔDA T test (p-value)
H3-b1	DA^R^<DA^F^	−1.808
		5.517**
		(0.000)
H3-b2	DA^B^<DA^A^	−2.215
		6.251**
		(0.000)
***H3-b1&2***	DA^R,B^<DA^F,A^	***−1.804***
		7.803**
		***(0.000)***

Superscript letters R, F, A, and B refer to XPs: Relaxation, Flow, Anxiety, and Boredom. ** p < 0.001, * p < 0.05.

**Table 5.** Student mean tests of DE comparisons.

Hypotheses	XPs under comparison	ΔDE T test (p-value)
H3-b3	DE^R^>DE^B^	0.911
		3.118*
		(0.002)
H3-b4	DE^F^>DE^A^	1.54
		4.911**
		(0.000)
***H3-b3&4***	DE^R,F^ > DE^B,A^	***0.991***
		4.770**
		***(0.000)***

Superscript letters R, F, A, and B refer to XPs: Relaxation, Flow, Anxiety, and Boredom. ** p < 0.001, * p < 0.05.

**Table 6.** Estimates of the gamer experience model.

	Dependent variable		
Explanatory variable	Boredom vs Flow	Relaxation vs Flow	Anxiety vs Flow
Intercept	2.710**	1.058	0.901
	(0.000)	(0.192)	(0.173)
DA	−0458**	−0.559**	0.0990
	(0.000)	(0.000)	(0.427)
DE	−0.303*	0.081	−0.557**
	(0.013)	(0.615)	(0.000)
Log LH	−183.43		
Wald χ^2^ test	58.71		
	(0.000)		
N	168		

p-values in parentheses (two-tail tests): ** p < 0.01, * p < 0.05

As for the subjective difficulty measures, DA and DE seem to be properly inferred from the enjoyment-based XP typology. Indeed, both the graph and the tests reported in Tables 4 and 5 support propositions P3-b linking the gaming experience to the two-dimensional challenge measure. As expected, the gamers experiencing anxiety or flow report significantly higher DA than others (boredom or relaxation). In the same way, on average, a significantly higher DE was reported by gamers in the flow and relaxation categories.

Moreover, the multi-layered enjoyment measures also improve the causal challenge-XP model, as shown in Table 6, below where DA and DE prove to be good predictors of the gaming experience. The theoretical framework of the multinomial probit model has each gamer I faced with J = 4 different gaming experiences at round R. The four gaming experience are Boredom (j = 1), Relaxation (j = 2), Anxiety (j = 3), and Flow (j = 4).

Table 6 provides the estimates and measures of fit of the multinomial probit model where the probability of experiencing (Boredom, Relaxation, Flow, or Anxiety) is estimated as a function of self-reported difficulty measures DA and DE, both ranging from 1 to 6.^13^ Flow is the reference gaming experience. Standard errors have been adjusted to account for panel data.

The results displayed in Table 6 fully bear out the causal model predicting the gamer experience based on her self-reported subjective difficulty measures DA and DE.

The multinomial probit estimates in Table 6 make it clear that, in accordance with Figure 2 and P3-a, high DA and DE decrease the probability of experiencing boredom rather than flow. DE being equal, a high DA decreases the likelihood of experiencing relaxation rather than flow, and DA being equal, a rise in DE reduces the probability of the anxiety state compared to the flow one.

#### Therefore, knowing DA and DE makes it possible to properly predict the gaming experience

These features justify the joint use of enjoyment in both the difficulty and concentration elicitation stages to rule out any ambiguity in the skill/challenge balance and to refine the estimation of the gaming experience. Moreover, data show that DE (whether high or low) makes it possible to predict the pleasant nature of the gamer experience (CE) in more than two-thirds of cases: enjoyable challenge DE_H_ (either low or high) leads to enjoyable experience CE_H_ in more than two-thirds of cases. In the same way, an unpleasant challenge (either low or high) results in an unpleasant gaming experience in more than two-thirds of cases.^14^

**Observation 4**: Subjective difficulty measures (DA and DE) and concentration ones (CA and CE) are in a one-to-one relationship linking challenge to XPs. Furthermore, regardless of both DA and CA, the two-layered enjoyment measures (CE and DE) highly correlate: a high DE ensuring a pleasant experience CE in two-thirds of cases. All these results prove the value of an approach based on enjoyment, which leads to a more accurate estimation of the link between challenge and gaming experience.

## Conclusion

Enjoyment is at the very heart of the motivation to play video games. As such, enjoyment needs to be central to the characterization of the player’s experience. The present approach broadens the standard view of enjoyment by accounting for the antagonistic tensions embedded in both the feelings of challenge and concentration.

Using an integrative approach of enjoyment, we characterize XPs based on a measure of concentration that incorporates the pleasure gained from being concentrated. A two-dimensional difficulty measure embedding a difficulty enjoyment component is then used to build enjoyment-based challenge mapping to better define the frontier of an enjoyable challenge, and to accurately assess the level of the challenge faced by a player. Eventually, the paper uncovers the relationship between XPs and challenge, and supports the whole enjoyment-based theoretical framework with enjoyment-based challenge mapping determining XPs.

These findings show how relevant this multi-layered enjoyment measure is to characterize the gaming experience (flow, anxiety, boredom, and relaxation), and to disentangle unbalanced challenging games: the data suggest that for a gaming experience to be self-rewarding, a high degree of concentration is not a necessary condition, or even a sufficient one. In the same way, by offering a finer assessment of the boundary of enjoyable difficulty, this work shows that both challenging and not challenging games can result in a pleasant state. Finally, altogether, concentration and challenge enjoyment measures provide useful tools to characterize the proximal conditions of the gaming experience.

Most importantly, difficulty enjoyment appears to prevail over difficulty assessment as it is more likely to ensure a pleasant (low or high attention-demanding) gaming experience: in more than two-thirds of cases, a pleasant challenge results in a pleasant experience (either relaxation or flow). Therefore, a sought out gaming experience could come down to being ‘enjoyably concentrated and challenged.’ Since the challenge enjoyment level much more than the challenge can ensure a positive (pleasant) game experience, game publishers should be aware of the importance of these enjoyment-based measures on the very nature of the game experience, and should be well advised to develop indicators to assess the twofold enjoyment dimension uncovered in this article.

This research paves the way for promising extensions. As Nacke and Lindley [26] who established a correlation between objective and self-reported measures, it could be interesting to examine whether physiological measures validate this causal model, and confirm both the typology of the players’ experiences and their link with enjoyment-based challenge mapping. This work should be done in the first place with physiological data from Levillain et al. [25] which were collected during this very same experiment.

As this extended approach of enjoyment provides tools to better understand and evaluate the mental states which gamers experience when performing tasks, it might also be relevant to apply this multi-layered enjoyment methodology to video games other than FPS, which are essentially immersive, and more broadly to tasks unrelated to video games.

## Notes

1. In their study, Orero et al. [2] also investigate players’ enjoyment/challenge relationship. However, in their paper, they use physiological signals collected during gameplay such as galvanic skin response (GSR), heart rate (HR), and respiration rate (RR) data. Their paper is an attempt to assess emotional experience during gameplay from physiological signals. Concentration and difficulty enjoyment-based measures were not examined.

2. Source: https://newzoo.com/resources/.

3. In their review, Meckler et al. [14] find that 82 papers out of 87 use subjective self-reporting in questionnaires with questions like ‘how fun was the game? How much did you enjoy the game’ etc.….

4. See [30] for a more detailed presentation of the experimental protocol.

5. For instance, questions related to the concentration feeling were: ‘How much concentration was required in this round?’ (Concentration-level Assessment, CA) and ‘To what extent did you enjoy this concentration level?’ (Concentration-level Enjoyment, CE).

6. DE is high (DEH) when DE>3, it is low (DEL) otherwise. The same applies to the DA variable.

7. The ‘Difficult’ round was chosen by almost all gamers (40 out of 41) as the most difficult one (difficulty comparative measure) and never as the least difficult. The ‘Introduction’ round was perceived as the least difficult one by 60% of gamers, and this proportion decreases with the objective difficulty level. None of the gamers rated it as the most difficult.

8. r_DA&DE_ = −0.16 (p-value = 0.041).

9. Moreover, the χ 2 test supports the independence hypothesis between DA and DE. χ² _DA&DE_ = 1.44 (p-value = 0.230).

10. From Table 2 (line (5)), 39/69 = 56% and 65/99 = 65% .

11. 76% = 100*(24 + 15 + 14 + 14)/(53 + 35) % and 79% = 100*(7 + 13 + 20 + 23)/(58 + 23).

12. Controlling for the standard deviations of the measures.

13. The dependent variable ‘gaming experience’ is categorical and unordered. The probabilities of the different gaming experiences sum up to 1.

14. P(CE_H_/DE_H_)≈67% and P(CE_L_/DE_L_)≈67%.

## References

[1] Csíkszentmihályi M. Flow: the psychology of optimal experience. New York: Harper and Row; 1990.

[2] Orero JO, Levillain F, Damez-Fontaine M, et al Assessing gameplay emotions from physiological signals: A fuzzy decision trees based model. International conference on kansei engineering and emotion research; 2010 3 pp. 1684–10.

[3] Abuhamdeh S, Csikszentmihalyi M The importance of challenge for the enjoyment of intrinsically motivated, goal-directed activities. Personality Soc Psychol Bull. 2012;38(3):317–330. 10.1177/0146167211427147 22067510

[4] Ryan RM, Rigby CS, Przybylski A The motivational pull of video games: A self-determination theory approach. Motiv Emot. 2006;30:347–363.

[5] Lucas K, Sherry JL Sex differences in video game play: A communication-based explanation. Communic Res. 2004;31(5):499–523.

[6] Chou C, Tsai MJ, Chou C, et al Gender differences in Taiwan high school students’ computer game playing. Comput Human Behav. 2007;23:812–824.

[7] Yee N Motivations for playing online games. Cyberpsychol Behav. 2006;9(6):772–775. 17201605 10.1089/cpb.2006.9.772

[8] Hsu CL, Lu HP, Hsu CL, et al Why do people play on-line games? An extended TAM with social influences and flow experience. Inf Manag. 2004;41:853–868.

[9] Fang X, Chan S, Brzezinski J, et al Development of an instrument to measure enjoyment of computer game play. Intl J Human - Comput Interact. 2010;26(9):868–886.

[10] Lin A, Gregor S, Ewing M Understanding the nature of online emotional experiences: a study of enjoyment as a web experience. In Proc. ICEC 2009; ACM; 2009 pp. 259–268.

[11] Klimmt C, Rizzo A, Vorderer P, et al Experimental evidence for suspense as determinant of video game enjoyment. Cyberpsychol Behav. 2009a;12(1):29–31. 19025464 10.1089/cpb.2008.0060

[12] Klimmt C, Hartman T, Frey A Effectance and control as determinants of video game enjoyment. Cyberpsychol Behav. 2007;10(6):845–847. 18085976 10.1089/cpb.2007.9942

[13] Klimmt C, Schmid H, Orthmann J Exploring the enjoyment of playing browser games. Cyberpsychol Behav. 2009b;12(2):231–234. 19260776 10.1089/cpb.2008.0128

[14] Mekler ED, Bopp JA, Tuch AN, et al A systematic review of quantitative studies on the enjoyment of digital entertainment games. In Proceedings of the 32nd annual ACM conference on Human factors in computing systems; ACM; 2014 4 pp. 927–936.

[15] Vorderer P, Klimmt C, Ritterfield U Enjoyment: at the heart of media entertainment. Commun Theory. 2004;14(4):388–408.

[16] Sweetser P, Wyeth P GameFlow: A model for evaluating player enjoyment in games. Comput Entertain. 2005;3(3):1–24.

[17] Jin SAA Toward integrative models of flow: effects of performance, skill, challenge, playfulness, and presence on flow in video games. J Broadcast Electron Media. 2012;56(2):169–186.

[18] Nacke LE, Grimshaw MN, Lindley CA More than a feeling: measurement of sonic user experience and psychophysiology in a first person shooter game. Interact Comput. 2009;22:336–342.

[19] Jennett C, Cox AL, Cairns P, et al Measuring and defining the experience of immersion in games. Int J Hum Comput Stud. 2008;66(9):641–661.

[20] Brockmyer JH, Fox CM, Curtiss KA, et al The development of the game engagement questionnaire: A measure of engagement in video game-playing. J Exp Soc Psychol. 2009;45:624–634.

[21] Boyle EA, Connolly TM, Hainey T, et al Engagement in digital entertainment games: A systematic review. Comput Human Behav. 2011;28(3):771–780.

[22] Jegers K Pervasive game flow: understanding player enjoyment in pervasive gaming. Comput Entertain. 2007;5(1):61–69.

[23] Cox A, Cairns P, Shah P, et al Not doing but thinking: the role of challenge in the gaming experience. Proceedings of the SIGCHI Conference on Human Factors in Computing Systems; ACM; 2012 pp. 79–88.

[24] IJsselsteijn W, Van Den Hoogen W, Klimmt C, et al Measuring the experience of digital game enjoyment. Proceedings of Measuring Behavior; Wageningen, Netherlands: Noldus Information Tecnology; 2008 pp. 88–89.

[25] Levillain F, Orero JO, Rifqi M, et al Characterizing player’s experience from physiological signals using fuzzy decision trees. In Computational Intelligence and Games (CIG) IEEE Symposium 2010; 75–82.

[26] Nacke L, Lindley CA Flow and immersion in first-person shooters: measuring the player’s gameplay experience. Proc. Future Play 2008; ACM; 2008 pp. 81–88.

[27] Trepte S, Reinecke L The pleasures of success: game-relatedefficacy experiences as a mediator between player performance and game enjoyment. Cyberpsychol Behav Soc Netw. 2011;14(9):555–557. 21342012 10.1089/cyber.2010.0358

[28] Ellis GD, Voelkl JE, Morris C, et al Measurement and analysis issues with explanation of variance in daily experience using the flow model. J Leisure Res. 1994;26(4):337–356.

[29] Nakamura J, Csikszentmihalyi M. The concept of flow In: Nakamura J, Csikszentmihalyi M, editors. Flow and the foundations of positive psychology. Netherlands: Springer; 2014 p. 239–263.

[30] Orero JO Modélisation de systèmes émotionnels à partir de signaux physiologiques et application dans la conception de jeux vidéo [Doctoral dissertation] Paris 6; 2011.

